# Triage and Ongoing Care for Critically Ill Patients in the Emergency Department: Results from a National Survey of Emergency Physicians

**DOI:** 10.5811/westjem.2019.11.43547

**Published:** 2020-02-24

**Authors:** Kusum S. Mathews, Sandra M. Rodriguez, Judith E. Nelson, Lynne D. Richardson

**Affiliations:** * Icahn School of Medicine at Mount Sinai, Division of Pulmonary, Critical Care and Sleep Medicine, Department of Medicine, New York, New York; † Icahn School of Medicine at Mount Sinai, Department of Emergency Medicine, New York, New York; ‡ Memorial Sloan Kettering Cancer Center, Weill Cornell College of Medicine, Departments of Medicine and Anesthesiology and Critical Care, New York, New York; § Icahn School of Medicine at Mount Sinai, Department of Population Health Science and Policy, New York, New York

## Abstract

**Introduction:**

We conducted a cross-sectional study at the Icahn School of Medicine at Mount Sinai to elicit emergency physician (EP) perceptions regarding intensive care unit (ICU) triage decisions and ongoing management for boarding of ICU patients in the emergency department (ED). We assessed factors influencing the disposition decision for critically ill patients in the ED to characterize EPs’ perceptions about ongoing critical care delivery in the ED while awaiting ICU admission.

**Methods:**

Through content expert review and pilot testing, we iteratively developed a 25-item written survey targeted to EPs, eliciting current ICU triage structure, opinions on factors influencing ICU admission decisions, and views on caring for critically ill patients “boarding” in the ED for >4–6 hours.

**Results:**

We approached 732 EPs at a large, national emergency medicine conference, achieving 93.6% response and completion rate, with 54% academic and 46% community participants. One-fifth reported having formal ICU admission criteria, although only 36.6% reported adherence. Common factors influencing EPs’ ICU triage decisions were illness severity (91.1%), ICU interventions needed (87.6%), and diagnosis (68.2%), while ICU bed availability (13.5%) and presence of other critically ill patients in ED (10.2%) were less or not important. While 72.1% reported frequently caring for ICU boarders, respondents identified high patient volume (61.3%) and inadequate support staffing (48.6%) as the most common challenges in caring for boarding ICU patients.

**Conclusion:**

Patient factors (eg, diagnosis, illness severity) were seen as more important than system factors (eg, bed availability) in triaging ED patients to the ICU. Boarding ICU patients is a common challenge for more than two-thirds of EPs, exacerbated by ED volume and staffing constraints.

## INTRODUCTION

The decision to triage critically ill patients to the intensive care unit (ICU) involves both objective and subjective patient-specific factors (e.g., co-morbidities, severity of illness, likelihood to benefit), as well as system factors (e.g., ICU bed availability, other waiting patients, availability of intermediate care beds).^1^ Many hospitals employ triage policies based on consensus recommendations for ICU admission focusing on patient factors (diagnosis, need for critical care interventions), especially during periods of ICU capacity strain,^2^ but these protocols are not consistently used even when available.^3^ While previous studies have surveyed emergency department (ED) and ICU providers about practice structures and guidelines, less is known about ICU triage decision-making in times of ICU bed shortage from the perspective of emergency physicians (EP).^3^,^4^

High demand for critical care services also led to significant increases in ED “boarding times”—ED lengths-of-stay greater than 4–6 hours— for critically ill patients awaiting ICU admission, complicating ED throughput and resource management.^4^,^5^ Critical care admission delays due to limited inpatient ICU bed availability have been associated with poorer patient outcomes.^6^ While critical care services is generally within the EP practice scope, less is known about their views on the delivery of ongoing ICU care in the ED setting.

The goals of this study were to identify factors contributing to ICU triage decisions and elicit EP perspectives on caring for critically ill patients with prolonged boarding times.

## METHODS

### Study Setting and Population

This study employed a survey administered to a cross-sectional convenience sample of EPs. Respondents were approached for participation at the Icahn School of Medicine at Mount Sinai over three consecutive days. Participants were eligible if they were either enrolled as an upper-level trainee in a US emergency medicine (EM) residency program (limited to postgraduate years 2–4 only) or were currently practicing as EPs at a clinical site in the US. Those who completed the survey were entered into a raffle to win monetary gift cards. Eligible participants were considered non-respondents if they declined to complete the survey. The study was determined to be exempt from review by the institutional review board at the authors’ institution, with dissemination of a research information sheet to all participants.

### Study Design and Measurements

A 25-item questionnaire-based survey with primarily closed-ended questions, was iteratively developed with content domains as follows: institutional structure for ICU admissions and ongoing management of ICU boarders, individual critical care triage practices and perspectives on how decisions are made,^1^ and caring for boarding ED patients awaiting ICU admission.^5^ Domains were selected after literature review and content development with ED and ICU physician feedback. Modifications were informed from cognitive interviews with 10 EPs addressing clinical sensibility (clarity, face validity, content validity, and utility) at the authors’ institution, followed by pilot testing to academic and community EPs at outside institutions. (See Appendix Survey for the final instrument.)

**Population Health Research Capsule**

What do we already know about this issue?

Intensive care unit (ICU) triage decisions involve various factors, with many of the “accepted” patients experiencing longer emergency department (ED) boarding times.

What was the research question?

This survey elicited emergency physician perspectives on ICU triage decisions and caring for those with long boarding times.

What was the major finding of the study?

Patient factors affect ICU triage more than ICU bed availability, despite increasing frequency of ED boarding.

How does this improve population health?

ED care for ICU boarders is affected by limited resources; more novel ways to improve throughput and deploy different care models may alleviate this growing problem.

We collected demographics, training background, and current practice information, including board certification status and completion of critical care fellowship training, if applicable. The survey included multiple-choice, Likert-type scales (five-point), answer selection with rankings in order of importance, and options for free-text completion. Respondents were advised to select one or multiple answers, as applicable to their practice setting.

### Analysis

Responses to Likert-type scale questions were coded as ordinal variables. Responses to the question on identification of factors affecting triage decisions were re-coded first as selected vs not selected and then, for those who provided ranking of their selections, factors were categorized based on the identified level of importance (Most/More important, Somewhat important, Less/Least important). The responses were described with univariate and bivariate analysis, stratified by university vs community practice setting, using chi square, Fisher’s exact, and independent t-testing, where appropriate. We performed analyses using SPSS Statistics, version 23 (IBM, Armonk, NY).

## RESULTS

A total of 732 attendees were approached for participation, with 685 eligible respondents completing the survey (93.6% response rate after excluding 18 surveys for non-US practice settings). Respondents were mostly attending physicians (78.1%), with representation from 47 states (Table 1). The majority reported caring for more than three critically ill patients per week (83.4%), and 72.1% reported that caring for boarding ICU patients was a frequent occurrence during their ED shifts.

### Main Results

Approximately one-fifth (n = 141) of respondents stated that their hospital had formal ICU admission criteria; of those, 60.3% reported consistent adherence with these guidelines. In-person ICU team consult for ICU admission was required in a minority of settings (n = 228/663, 33.3%), more commonly seen in university over community settings (45.6 vs 21.6%, p<0.001). While the ED team was identified as the final triage decision-maker for ICU admission in community hospitals (ED 54.3 vs ICU 19.9%), the ICU team finalized the ICU admission decisions more often in university hospitals (ED 42.9 vs ICU 46.8%, p<0.001). A hospitalist team (n = 67/663, 9.8%) or joint decision-making structure (n = 35/663, 5.1%) for triaging ICU admissions was infrequently reported, regardless of practice setting.

Factors identified as contributing to the EP’s ICU triage decision included severity of illness (91.1%), need for critical care interventions (87.6), and diagnosis (68.2%), with minimal differences between university or community settings. (See Table 2 for respondent-identified factors; Appendix Table for respondent-ranked factors.) Only 23.2–35.3% of respondents emphasized age and prehospital status as contributory to the ICU admission decision, although the perception that patients would likely benefit from critical care intervention was identified as important by 56% of respondents. System factors related to ICU demand (both inpatients and others in the ED waiting for ICU admission) were least important to EPs (10.2–13.5%). Survey respondents reported that it was a common experience for patients to be denied ICU admission by the primary ICU team in their hospital (n = 255/531, 48.0%) reported denials as an “always, often, or sometimes” occurrence), with given reasons by ICU team being more often due to limited ICU bed availability (n = 153/647, 23.6%) and patient suitability for an intermediate care unit as an alternative to an ICU (n = 440/647, 68.0%).

ICU boarding time greater than 4–6 hours was frequently observed (71.5%), with the ED remaining the primary team while boarding (50.8%). The majority (73.7%) reported that these patients typically remained in the ED until an ICU bed opened; temporary transfer to other units (eg, intermediate care unit, post-ambulatory care unit, overflow units, etc) was uncommon. Respondents identified high patient load per provider (64.8%), high overall ED volume (51.5%), and insufficient support staff (51.4%) as the primary barriers to ongoing care, while personal discomfort with caring for boarding ICU patients (18.8%) was less common. Those practicing at community hospitals more often identified staffing and resource constraints as hindering high-quality care delivery to ICU boarders (Figure). Communication with the ICU team was also rated as only sometimes to rarely helpful by over one-third of respondents (n = 235/655, 35.9%). Most agreed with the statement that the ED team should not be required to manage ICU boarders on their own (n = 573/648, 88.4%), but only 38.4% definitively stated it should be the primary responsibility of the ICU or inpatient teams. One-quarter of respondents (n = 167/659) stated that their EDs employed board-certified ED intensivists, with this being a more frequent occurrence in university over community settings (37.6 versus 11.4%, p<0.001).

## DISCUSSION

Our study demonstrates that ED triage decisions are more informed by the patient’s acute presentation, than by factors associated with the perceived risks and benefits of ICU care. In contrast to past studies, which identify ICU bed availability and consideration of other waiting patients as affecting ICU triage decisions,^1^,^6^ our study also demonstrates that these system constraints appear to factor less into EP decision-making. While past studies have assessed triage decision-making from ICU providers’ perspective,^7^ to our knowledge, this is the first study to elicit EP perceptions about ICU triage decisions and care for critically ill patients “boarding” in the ED. While our study ascertained the relative irrelevance of the system factors to ED decision-making around ICU triage, respondents commonly received ICU denials for their patients, with the perception that ICU capacity does play a role in ICU team decision-making. Similar to ICU physician surveys, EPs highlight that established institutional triage criteria and protocols are infrequently applied.^8^

Our results also support the concern for the growing workload associated with the increasing number of boarding ICU patients in the ED.^4^ The majority of survey respondents, regardless of practice setting, reported that patients remain in the ED until ICU beds become available, and that the ED team is primarily responsible for the ongoing critical care management. Delays in ICU admission have been associated with poorer outcomes for critically ill ED patients,^6^,^9^ but as our survey respondents identified, high volume weighs heavily into the ability and capability of EPs to optimally take care of these patients. Crowding and inpatient ED boarding are associated with lower likelihood of receiving best-practice recommendations for various critical diagnoses, including sepsis and myocardial infarction. Improvements in hospital-wide throughput are needed to alleviate inpatient bottlenecks felt by the ED.

With fixed ICU availability and a growing number of ICU boarders in the ED, adaptation and evolution of the traditional critical care delivery model (previously limited to care by ED or inpatient ICU teams) are already being developed to address concerns identified in this survey. Resource and staffing limitations were pinpointed as significant constraints to providing optimal care for critically ill patients while boarding. Many EDs may not have access to flexible nursing pools to maintain ICU-level staffing ratios two patients to one nurse. Additionally, our study supports the fact that communication between the ED and ICU teams has room for improvement. Newer models of ED-based intensive care units or flexible mobile ICU teams may prove helpful in improving collaboration between teams, alleviating some of the workload burden, and sustain high-quality critical care delivery until transfer to inpatient ICU bed occurs.^10^ Although our survey identified a fair number of practice settings employing ED intensivists, advanced critical care training for the EP is still in the minority.^11^ These alternative care models, while highly variable in structure, provide more specialized opportunities for the critical care medicine-trained EP and support for both the EM and ICU inpatient teams and potentially improved outcomes for critically ill ED boarding patients.^12^

## LIMITATIONS

Limitations of this study include its closed-ended survey design, convenience sampling, and respondent and recall bias for boarding frequency and factors impacting decision-making, precluding a deeper understanding of triage complexity and boarding ICU patient care. Practice locations were not identified, allowing for the possibility of multiple responses from the same institution. However, large response rates with national representation provide confirmation of the system-related challenges associated with boarding commonly felt by many EPs. Triage decision-making, with comparisons between emergency and ICU physicians, warrants further investigation and may be better elicited through interviews or focus groups, and/or a mixed methods approach.

## CONCLUSION

In this nationally representative survey of EPs, patient-related factors were seen as more important than system factors (eg, bed availability) in triaging ED patients to the ICU, despite the high frequency of prolonged boarding across practice settings. Caring for boarding ICU patients is affected by high ED volume and staffing constraints, suggesting that more innovative ways to improve ICU throughput and employ alternative critical care delivery models may help to alleviate this growing problem.

## Supplementary Information





## Figures and Tables

**Figure:**
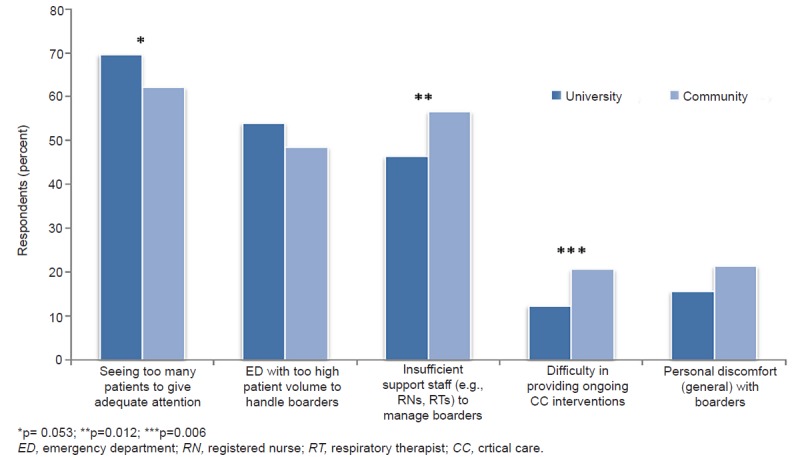
Attitudes of emergency medicince physicians toward caring for critically ill ED patients wih prolonged ED boarding times (greater than 4–6 hours), stratified by hospital setting. *p= 0.053; **p=0.012; ***p=0.006 *ED*, emergency department; *RN*, registered nurse; *RT*, respiratory therapist; *CC*, crtical care.

**Table 1:** Characteristics of Emergency Medicine (EM) physician respondents, stratified by those who primarily work in university/teaching versus community hospitals.

Characteristics	Respondents (N=685)	University/teaching hospital† (N=342)	Community hospital† (N=286)
Gender (%)**			
Male	461/684 (67.3)	215 (62.9)	208 (72.7)
Female	223/684 (32.6)	127 (37.1)	78 (27.3)
Age (mean ± SD) ***	41.9 ± 11.7	39.2 ± 11.1	44.2 ± 11.2
Geographic distribution (%)**			
Northeast	182/615 (26.6)	116 (33.9)	57 (19.9)
Midwest	180/615 (26.3)	87 (25.4)	81 (28.3)
South	156/615 (22.8)	70 (20.5)	75 (26.2)
West	97/615 (14.2)	39 (11.4)	51 (17.8)
Level of experience (%)			
Current trainee‡	143/654 (21.9)	108 (31.6)	25 (8.7)
Attending physician	511/654 (78.1)	221 (64.6)	249 (87.1)
Years in practice (Median, IQR)*	10 (4–20)	9 (3–18.5)	11 (5–22)
U.S. Board certified in EM	453/613 (66.1)	185 (84.9)	215 (87.8)
Critical care (CC) fellowship	12/659 (1.8)	6 (1.8)	4 (1.4)
Practice setting (%)			
University/teaching	342 (49.9)		
Community	286 (41.8)		
Veterans Affairs	5 (0.7)		
Managed care hospital	6 (0.9)		
Multiple settings	27 (3.9)		
Other/not specified	19 (2.8)		

* p<0.05,

** p<0.01;

*** p<0.001;

† Limited to those with identification of the primary practice setting as either university or community hospital. All numbers listed in parentheses are a percentage of the total within the category of either university or community hospital setting.

‡ Current trainees are upper-level EM residents, post-graduate years 2–4.

*SD*, standard deviation; *CC*, critical care; *EM*, emergency medicine; *IQR*, interquartile range; *SD*, standard deviation.

**Table 2:** Identified factors affecting Emergency Medicine physician ICU triage and admission decision-making,* stratified by hospital setting.

Factors	Total (N=638)**	University/Teaching Hospital† (n=317/589)	Community Hospital† (n=272/589)
Patient-related factors (%)			
Acuity/severity of illness	581 (91.1)	289 (91.2)	249 (91.5)
CC intervention needed	559 (87.6)	282 (89.0)	238 (87.5)
CC diagnosis	435 (68.2)	208 (65.6)	198 (72.8)
Likelihood to benefit	357 (56.0)	182 (57.4)	149 (54.8)
Age and/or co-morbidities	225 (35.3)	108 (34.1)	104 (38.2)
Pre-existing goals of care	221 (34.6)	113 (35.6)	92 (33.8)
Pre-hospital quality of life	148 (23.2)	73 (23.0)	63 (23.2)
Hospital/system-related factors (%)			
ICU team input	203 (31.8)	109 (34.4)	81 (29.8)
Hospital’s admission criteria‡	98 (15.4)	59 (18.6)	32 (11.8)
ICU bed availability	86 (13.5)	45 (14.2)	34 (12.5)
Step-down bed availability	70 (11.0)	35 (11.0)	27 (9.9)
Other CC patients in ED	65 (10.2)	38 (12.0)	23 (8.5)

* Identified factors include all selected and/or positively ranked responses: Yes; Most, Very, or Moderately important

** Of the total 685 survey respondents, 638 (97.0%) answered this question. Total includes 49 respondents who identified multiple clinical sites (n=23), Veterans Affairs hospital (n=5), Managed Care Hospitals (n=5), and Other/Non-specified (n=16), as their primary practice setting.

† Question Responses from survey participants who identified University/Teaching Hospital (n=317/342) or Community Hospitals (n=272/286) as their primary practice setting are included in the second and third columns respectively. All numbers in parentheses reflect the percentage of the total of respondents from university or community settings.

‡ p=0.022

*CC*, critical care; *ICU*, intensive care unit; *ED*, emergency department.

## References

[1] Gopalan PD, Pershad S (2018). Decision-making in ICU: A systematic review of factors considered important by ICU clinician decisionmakers with regard to ICU triage decisions. J Crit Care.

[2] Nates JL, Nunnally M, Kleinpell R (2016). ICU admission, discharge, and triage guidelines: a framework to enhance clinical operations, development of institutional policies, and further research. Crit Care Med.

[3] Walter KL, Siegler M, Hall JB (2008). How decisions are made to admit patients to medical intensive care units (MICUs): a survey of MICU directors at academic medical centers across the United States. Crit Care Med.

[4] Magid DJ, Sullivan AF, Cleary PD (2009). The safety of emergency care systems: results of a survey of clinicians in 65 US emergency departments. Ann Emerg Med.

[5] American College of Emergency Physicians (2017). Boarding of admitted and intensive care patients in the emergency department [press release].

[6] Mathews KS, Durst MS, Vargas-Torres C (2018). Effect of emergency department and ICU occupancy on admission decisions and outcomes for critically ill patients. Crit Care Med.

[7] Kohn R, Rubenfeld GD, Levy MM (2011). Rule of rescue or the good of the many? An analysis of physicians’ and nurses’ preferences for allocating ICU beds. Intensive Care Med.

[8] Ward NS, Teno JM, Curtis JR (2008). Perceptions of cost constraints, resource limitations, and rationing in United States intensive care units: results of a national survey. Crit Care Med.

[9] Groenland CNL, Termorshuizen F, Rietdijk WJR (2019). Emergeny department to ICU time is associated with hospital mortality: a registry analysis of 14,788 patients from six university hospitals in the Netherlands. Crit Care Med.

[10] Leibner E, Spiegel R, His C (2019). Anatomy of resuscitative care unit: expanding the borders of traditional intensive care units. Emerg Med J.

[11] Napolitano LM, Rajajee V, Gunnerson KJ (2018). Physician training in critical care in the United States: Update 2018. J Trauma Acute Care Surg.

[12] Gunnerson KJ, Bassin BS, Havey RA (2019). Association of an emergency department-based intensive care unit with survival and inpatient intensive care unit admissions. JAMA Netw Open.

