# Supplementary material for: Triage and Ongoing Care for Critically Ill Patients in the Emergency Department: Results from a National Survey of Emergency Physicians
Source: West J Emerg Med. 2020 Feb 24;21(2):330–5. doi: 10.5811/westjem.2019.11.43547 (PMC7081882; doi:10.5811/westjem.2019.11.43547)
Supplement: Supplementary file 1 [file wjem-21-330-s001.docx]

**Appendix: Survey**

**Critical Care practices in the Emergency Department**

**The purpose of this questionnaire is to assess Emergency Physicians’ perceptions of critical care provision in the Emergency Department (ED), decision-making around Intensive Care Unit (ICU) admission, and the relationship between the ED and ICU providers. All answers are completely anonymous and will be aggregated for later analysis. Thank you for participating!**

**Please complete the following general demographic questions:**

1. **Gender**: 🞎 Male 🞎 Female
2. **Age (years):** ____
3. **In what YEAR, did you (or will you) complete your residency?** _____________
4. **Are you currently U.S. board-certified in Emergency Medicine?** 🞎 Yes 🞎 No
5. **In which type of setting is your primary practice?**
   🞎 University or teaching hospital
   🞎 Community
   🞎 Managed care hospital
   🞎 Veterans
   🞎 Other ______________
6. **In what state is your primary practice?** ­­­­­­­­­_________
7. **Have you completed any formal critical care fellowship training?** 🞎 Yes 🞎 No

**7a: If yes to Q7, what type?**
🞎 Internal Medicine-Critical Care

🞎 Surgical Critical Care

🞎 Anaesthesia Critical Care Medicine

🞎 Other ___________________________

**7b: If yes to Q7, are you currently U.S. board-certified in Critical Care Medicine?** 🞎 Yes 🞎 No

**For the following series of questions, please focus on ED patients seen in the last ONE YEAR who required admission to a *MEDICAL ICU*. Choose the answer most reflecting your hospital’s current practice.**

1. **Does your hospital require an IN-PERSON assessment by an ICU team member for critically ill patients in the ED, prior to ICU admission?** 🞎 Yes 🞎 No 🞎 Unsure
2. **Who usually makes the final decision to admit a patient to your institution’s Medical ICU?**

🞎 ED team 🞎 ICU team 🞎 Hospitalist team 🞎 Other ________________

1. **Does your hospital have a set of WRITTEN CRITERIA FOR ICU ADMISSION?**

🞎 Yes 🞎 No 🞎 Unsure

**3a. If yes to Q3, how often are these criteria used to determine ICU admission?**

🞎 Always 🞎 Often 🞎 Sometimes 🞎 Rarely 🞎 Never

1. **When a critically ill patient is accepted for ICU admission, which team is responsible for ongoing clinical management until physical departure from the ED?**

🞎 ED team 🞎 ICU team 🞎 Hospitalist team 🞎 Other ________________

1. **When the Medical ICU is full at your hospital, critically ill patients who require ICU admission are usually managed in which location?**

🞎 ED until Medical ICU bed available

🞎 Intermediate care unit (e.g., Step-Down Unit) or PACU if bed available

🞎 Another ICU (e.g., Surgical or Neuro ICU) if bed available

🞎 Other ______________

1. **After initial resuscitation/stabilization, does your ED have a dedicated internal area/unit (e.g., Resuscitation Room, ED-based ICU, etc. ) for critically ill patients to remain while waiting for transfer to an in-patient ICU?** 🞎 Yes 🞎 No 🞎 Unsure

**6a. If yes to Q6, how many beds are within that area/unit?** ____________

1. **Do you currently have emergency physicians—who are BOARD-CERTIFIED in critical care—working in your ED?**

🞎 Yes 🞎 No 🞎 Unsure

**For the following series of questions, please focus on ED patients that YOU treat, who require admission to a *MEDICAL ICU*. Choose answers that most reflect YOUR current practice and opinions.**

1. **On average, how often do YOU treat patients in the ED, requiring Medical ICU services?**

🞎 <1-3 patients/week 🞎 3-6 patients/week 🞎 7-10 patients/week 🞎 >10 patients/week

1. **Which factors play a role in your decision that a patient needs ICU-level care?
   (Rank all that apply: 1 = Most important to 5 = Least important.)**

__ Patient’s diagnosis

__ Critical care intervention(s) needed

__ Acuity or severity of illness

__ Patient’s goals of care

__ Patient age and/or co-morbidities

__ Patient’s pre-hospital quality of life
__ Likelihood to benefit from critical care services

__ ICU bed availability

__ Intermediate care unit
 (e.g., Step-Down Unit) bed availability

__ Other critically ill patients in the ED

__ Input from the ICU team

__ Hospital-issued admission criteria

__ Other ___________________________

1. **How would you describe the communication you have with ICU teams about critically ill patients still in ED?**

🞎 Always helpful 🞎 Usually helpful 🞎 Sometimes helpful 🞎 Rarely helpful 🞎 Never helpful

1. **If your patient is DENIED admission by accepting ICU team, what reason(s) is/are given? Check all that apply:**

🞎 Can be managed on floor/Step-Down

🞎 Lower acuity or severity of illness

🞎 Unlikely to benefit from ICU services

🞎 N/A: ICU cannot “reject” ED admissions

🞎 Goals of care not consistent with ICU care

🞎 No ICU bed availability

🞎 Other ___________________________

**4a. If patients are DENIED admission by the ICU team at your institution, how often does this happen?**

🞎 Always 🞎 Often 🞎 Sometimes 🞎 Rarely 🞎 Never

1. **How often do patients admitted to an ICU remain in the ED for >4-6 hours awaiting transfer to an in-patient unit (a.k.a. “Boarding Times”)?**

🞎 Always 🞎 Often 🞎 Sometimes 🞎 Rarely 🞎 Never

1. **How would you describe your perceptions about critically ill patients with Boarding Times > 4-6 hours?
   Check all that apply.**

🞎 I see no difficulty caring for these patients in the ED as long as necessary.

🞎 I am qualified to care for these patients, but seeing too many other patients to give them adequate attention.

🞎 I am not comfortable caring for these patients for longer than 4-6 hours.

🞎 My ED has too high patient volume (e.g., ED crowding) to handle these patients for longer than 4-6 hours.

🞎 My ED does not have enough support staff (e.g., nurses, respiratory therapists, etc.) to safely manage these patients for longer than 4-6 hours.

🞎 My ED is unable to provide ongoing ICU-level interventions (e.g., hemodynamic monitoring, complex ventilator or vasopressor management) for longer than 4-6 hours.

🞎 An ED-staffed team should be required to manage these patients while still in the ED.

🞎 An ICU team (or another non-ED team) should be required to manage these patients while still in the ED.

🞎 Other ____________________________
