# Supplementary material for: Triage and Ongoing Care for Critically Ill Patients in the Emergency Department: Results from a National Survey of Emergency Physicians
Source: West J Emerg Med. 2020 Feb 24;21(2):330–5. doi: 10.5811/westjem.2019.11.43547 (PMC7081882; doi:10.5811/westjem.2019.11.43547)
Supplement: Supplementary file 2 [file wjem-21-330-s002.docx]

**Appendix Table:** Factors affecting Emergency Medicine physicians’ decision-making around ICU triage and admission, ranked responses*

|  | Total Ranked  Responses | Most/More Important | Somewhat Important | Less/Least Important |
| --- | --- | --- | --- | --- |
| **Patient-related factors (%)** |  |  |  |  |
| - Acuity/severity of illness - CC intervention needed - CC diagnosis | 515  499  453 | 454 (88.2)  434 (87.0)  259 (57.2) | 43 (8.3)  42 (8.4)  99 (21.9) | 18 (3.5)  23 (4.6)  95 (21.0) |
| - Likelihood to benefit - Age and/or co-morbidities - Pre-existing goals of care - Pre-hospital quality of life | 418  326  312  226 | 218 (52.2)  95 (29.1)  112 (35.9)  64 (28.3) | 88 (21.1)  97 (29.8)  78 (25.0)  65 (28.7) | 112 (26.8)  134 (41.1)  122 (39.1)  97 (42.9) |
| **Hospital/System-related factors (%)** |  |  |  |  |
| - ICU team input - Hospital’s admission criteria | 273  216 | 105 (38.5)  54 (25.0) | 71 (26.0)  31 (14.4) | 97 (35.5)  131 (60.6) |
| - ICU bed availability - Step-Down bed availability | 252  195 | 46 (18.2)  23 (11.8) | 20 (7.9)  38 (19.5) | 186 (73.8)  134 (68.7) |
| - Other CC patients in ED | 216 | 27 (12.5) | 30 (13.9) | 139 (64.4) |

*CC,* critical care*.*

*Excludes responses with checkmarks on written survey.
